# Abstracts

**DOI:** 10.1155/S1463924684000134

**Published:** 1984


Journal of Automatic Chemistry, Volume 6, Number 2 (April–June 1984), pages 62–66

### Abstracts

### Page 67

### The Perkin-Elmer approach to computer-aided chemistry: an overview of technological trends in laboratory computing

S. A. Reber and J. P. Coates

At Perkin-Elmer, the term 'computer-aided chemistry' means the application of computers—of any size—to the solution of a chemical problem. In other words, the computer is a tool which handles data so that the chemist is freed to do chemistry.

It is possible to comprehend the future technological trends for this extremely important tool only by understanding the range of its applications in laboratories, and so this paper begins by classifying laboratories according to the types of problem-solving they do. For instance, a quality-control laboratory is confronted with quite different problems than is an R&D laboratory. Within this context, four important technological trends are considered. First, the subject of hardware development is treated, and the future roles of microprocessors, microcomputers (data stations), and minicomputers, which comprise the three hardware levels of Perkin-Elmer's Computer-Aided Chemistry scheme, are contemplated. At the third (minicomputer) level, the major issue is the future importance of centralized versus distributed data-processing. Centralized processing corresponds to the 'laboratory automation' product which is already well-known in the chromatography world; the Perkin-Elmer embodiment runs on a P-E Model 3210 superminicomputer, and is called C/LAS ('Chromatography Laboratory Automation System').

In a laboratory, the term 'distributed data processing' usually refers to the performance of data reduction on microcomputers. As microcomputer prices fall, and as the general understanding of micros among the general population increases, the use of micros for general data reduction will continue to rise.

The second subject of this paper i[illegible] the emergence of packaged (turnkey) applications software for analytical chemistry as, perhaps, the key technological development because its existence is the key to the use of micros.

The growing power of micros will extend their use into areas where minis were previously required; therefore, new software packages will be developed to serve these applications.

Several important tools will also foster the proliferation of high-level software for analytical applications. One category of these 'tools' may be termed 'ergonomics', since it pertains to the use of improved user interactions with the computer. A second category is the trend toward writing software in high-level languages, due to greater computer power and standard operating systems. The third category is the incorporation of better graphical techniques.

Also lying within the realm of microcomputer applications software is the rapidly increasing use of micros for ancillary functions, like word processing, spread sheet calculations, and data management.

The third key area discussed in this paper is laboratory information management. This subject goes far beyond the ordinary meaning of data-base management. While it requires data-base management as a tool, it really means the development of applications software which places the proper information at the fingertips of all laboratory personnel: clerks, secretaries, technicians, chemists, section supervisors, and laboratory managers, so that proper decisions are made and resources can be drawn.

Finally, important progress is being made in the area of communications, specifically, local and global networking, and the impact of this technology is discussed. Particularly noteworthy is the subject of local area networking and its significance to the distributed environment discussed earlier.

### Computer aided chemistry tel que le voit Perkin-Elmer. Revue de tendences techniques dans l'utilisation d'ordinateurs au laboratoire

S. A. Reber and J. P. Coates

Chez Perkin Elmer l'expression 'Computer-aided chemistry' veut dire l'application d'ordinateur de n'importe quelle taille à la solution d'un problème chimique.

Cette publication commence donc en classifiant les laboratoires selon le genre de problèmes qu'ils ont à résoudre. Dans ce contexte, quatre tendences technologiques importantes doivent être considérées. Premièrement, le développement du matériel est traité, et le rôle future des microprocesseurs, microordinateurs (stations de données) et des miniordinateurs est discuté. Au troisième niveau (miniordinateur), le problème principal est l'importance dans le future du traitement de données centralisé ou local. Le traitement centralisé correspond à l'automation de laboratoire déjà bien connue dans le monde de la chromatographie.

Dans un laboratoire, l'expression 'traitement de données local' décrit d'habitude la réduction de données par microordinateurs.

Le deuxième sujet de cette publication est l'apparition de logiciels d'applications complets pour la chimie analytique qui est probablement le développement technologique principal étant la clef pour l'utilisation des micros.

La nouvelle puissance des micros étendra leur utilisation dans des domaines qui, dans le temps, exigeaient des minis; donc de nouveaux logiciels seront développés pour servir ces applications.

Différents outils importants ont également aidé la prolifération de logiciels au niveau pour applications analytiques. Une catégorie des ces outils peut être appelée ergonomique, car leur but principal est d'améliorer l'interaction avec l'ordinateur. Une deuxième catégorie est la tendence vers des logiciels écrits en langages à haut niveau. La troisième catégorie est l'introduction de techniques graphiques meilleures.

Egalement, dans le domaine des nouveaux logiciels d'applications pour microordinateurs sont l'utilisation augmentente des micros pour des fonctions auxiliaires tels que le traitement de text, rédaction de comptes-rendus et la gestion de données.

Le troisième domaine important discuté dans cette publication est la gestion d'informations de laboratoire. Tandis que'il utilise le traitement de données comme outil, la signification réelle est le développement d'un logiciel d'application qui place les informations nécessaires à toutes les personnes engagés au laboratoire: secrétaires, techniciens, chimistes, chefs de laboratoire.

Finalement, des progrès importants ont été faits dans le domaine de communication, spécialement en réseaux locaux et globaux. Particulièrement important nous semble le sujet des réseaux locaux et sa signification pour la distribution de données discutées au paravant.

### Computer Aided Chemistry: Eine Übersicht technologischer Trends im Rechnereinsatz für das Labor

S. A. Reber and J. P. Coates

Der von Perkin-Elmer geprägte Ausdruck 'Computer-aided chemistry' umfasst die Anwendung von Rechnern jeder Art auf die Lösung chemischer Probleme. Die Arbeit beginnt zunächst mit einer Klassifizierung der Laboratorien nach der Art der Probleme, die gelöst werden. In diesem Zusammenhang werden vier wichtige technologische Trends betrachtet. Erstens werden die Entwicklungen der Hardware diskutiert, und die künftige Rolle von Mikrocomputer und Minicomputer betrachtet. Auf der obersten Stufe ist eine Hauptfrage, die der zentralen Lösung im Vergleich zur verteilten Lösung. Die zentrale Lösung entspricht der Laborautomation wie sie bereits in der Chromatographie gut bekannt ist. Im Labor bedeutet der Ausdruck 'verteilte Datenverarbeitung' die Datenerfassung und Vorverarbeitung mit einem Mikrocomputer.

Das zweite Thema ist das Auftauchen von betriebsbereiten Software-Paketen für die chemische Analytik. Das ist für die Benützung von Mikrocomputern die entscheidende technologische Entwicklung. Mikrocomputer werden künftig auch Funktionen von Minicomputern übernehmen. Entsprechend werden neue Software-Pakete benötigt. Verschiedene Software-Werkzeuge werden die Verbreitung von solchen Paketen für analytische Anwendungen beschleunigen. Eine Kategorie namens Ergonomie umfasst die verbesserte Interaktion zwischen Benützer und Rechner. Eine zweite betrifft die Verwendung von highlevel Sprachen zum Erstellen der Software. Die dritte ist der Einschluss graphischer Verfahren. Daneben können die Mikrocomputer auch für zusätzliche Funktionen wie Textverarbeitung, Tabellenkalkulationen und Datenverwaltung eingesetzt werden.

Das dritte Thema betrifft die Laborinformationsverwaltung. Neben einem Datenverwaltungssystem wird zusätzliche Software notwendig um die Informationen dem gesamten Laborpersonal zugänglich zu machen: Büroangestellte, Sekretärinnen, Laboranten, Chemiker, Laborchefs und Bereichsleiter.

Schliesslich ist zur Zeit ein rascher Fortschritt in der Kommunikation zu verzeichnen, speziell lokale und globale Netzwerke. Speziell bemerkenswert ist das Thema der lokalen Netzwerke in seiner Bedeutung für die verteilte Struktur wie sie weiter oben angeführt wird.

### Page 74

### A computerized automatic apparatus for determination of mercury in biological samples

Ö. Einarsson *et al.*

A computerized automatic apparatus for determination of mercury in biological samples (mainly urine and blood) and aqueous solutions has been constructed. Its working principle is flameless atomic absorption after liberation of mercury by reduction with $Sn^{2+}$. The brain of the instrument is a desk computer (Compucorp Statistician), which steers the pumps and valves, the sample changer etc. The working scheme is easily altered by changing the computer program. The analysis time of each sample is 2·5–3 min, and the detection limit is 0·2–0·3 ng Hg per sample. The coefficient of variation was found to be 1·2% for aqueous solutions and 3·3% for blood samples. 0·2 ml samples of both urine and blood are used. The samples are wet digested before being introduced into the apparatus. The detection limit covers the mercury content of samples from occupationally unexposed persons.

Absorbance readings are made either by a printer or by a recorder. These readings were compared to peak area readings, which were less dependent on the matrix of the sample, but a variation of about 10% in sensitivity is acceptable with exposure control analyses, and the more convenient printer absorbance readings are used for routine investigations.

The variation in sensitivity during one year's use was 6·60% for urine ($N=37$) and 6·93% for blood ($N=54$). In absorbance units, the sensitivity was more than 20% lower for blood than for urine. This can be attributed to the higher viscosity of digested blood samples, as well as to a small loss of mercury during the digestion of blood samples.

### Un instrument automatique pour la détermination du mercure dans les échantillons biologiques

Ö. Einarsson *et al.*

Un instrument automatique controlé par ordinateur pour la détermination du mercure dans les échantillons biologiques (surtout urine et sang) et dans des solutions aqueuses a été construit. Le principe de travail est l'absorption atomique sans flamme après libération du mercure par réduction avec $Sn^{2+}$. Le cerveau de l'instrument est un miniordinateur (Compucorp Statistician), qui contrôle les pompes et les vannes, l'échantillonnage etc. La séquence d'opérations est facilement changée en modifiant le programme de l'ordinateur. Le temps d'analyse pour chaque échantillon est de 2,5–3 min et la limite de détection est de 0,2–0,3 ng Hg par échantillon. Le coéfficient de variation était de 1,2% pour solutions aqueuses et 3,3% pour échantillon de sang. 0,2 ml d'urine et de sang sont utilisés comme échantillon. Les échantillons sont minéralisés par voie humide avant l'introduction dans l'instrument. La limite de détection couvre également la concentration de mercure d'échantillons de personnes non-exposées aux mercure.

Les mesures d'absorption sont faites soit par imprimante ou par enregistreur. Ces mesures sont comparées aux mesures de surfaces de pics, qui dépendent moins de la matrice de l'échantillon, mais une variation d'environ 10% en sensibilité est acceptable pour des analyses de contrôle d'exposition, et les mesures d'absorption pratique par imprimante sont utilisées pour les contrôles de routine.

Les variations de sensibilité durant une année étaient de 6,60% pour l'urine ($N=37$) et 6,93% pour le sang ($N=54$). En unités d'absorption la sensibilité était environ 20% plus basse pour le sang que pour l'urine. Ceci peut être attribué à la viscosité plus élevée des échantillons de sang minéralisés, ainsi qu'à la petite perte de mercure pendant la minéralisation des échantillons de sang.

### Ein automatisches Gerät mit Computer zur Bestimmung von Quecksilber in biologischen Proben

Ö. Einarsson *et al.*

Es ist ein automatisches Gerät mit Computer zur Bestimmung von Quecksilber in biologischen Proben (hauptsächlich Urin und Blut) und in wässrigen Lösungen entwickelt worden. Das Arbeitsprinzip ist die flammenlose Atomabsorption nach der Freisetzung von Quecksilber durch Reduktion mit $Sn^{2+}$ Die Intelligenz des Gerätes besteht aus einem Tischrechner (Compucorp Statistician), der die Pumpen, die Ventile, den Probenwechsler etc. steuert. Das Ablaufschema kann durch Änderung des Computerprogramms leicht geändert werden. Die Analysendauer für eine Probe beträgt 2·5–3 Minuten, und die Detektionsgrenze beträgt 0,2–0,3 ng Hg pro Probe. Es wurde ein Variationskoeffizient von 1,2% für wässrige Lösungen und von 3,3% für Blutproben gefunden. Die verwendete Probenmenge für Urin und Blut betrug 0·2 ml. Die Proben wurden vor der Einführung in die Apparatur nass aufgeschlossen. Die Detektionsgrenze deckt den Quecksilbergehalt der Proben von arbeitsplatzmässig nicht exponierten Personen ab.

Die Absorptionsmessung wird entweder mit einem Printer oder einem Schreiber erfasst. Diese Messungen wurden mit Bestimmungen der Fläche unter der Absorptionskurve verglichen, welche weniger von der Matrix Probe abhangen. Eine Variation der Empfindlichkeit von etwa 10% kann aber für Kontaminations-Kontrollanalysen toleriert werden, und die praktischere Erfassung der Absorption mit dem Printer wird deshalb für Routineuntersuchungen verwendet.

Die Variation der Empfindlichkeit während eines Jahres betrug 6,60% für Urin ($N = 37$) und 6,93% für Blut ($N = 54$). In Absorptionseinheiten war die Empfindlichkeit in Blut, im Vergleich zu Urin, mehr als 20% niedriger. Das kann der höheren Viskosität der aufgeschlossenen Blutproben als auch einem kleinen Verlust von Quecksilber während des Aufschlusses der Blutproben zugeordnet werden.

### Page 80

### An automated system of sample analysis for a total carbon analyser

L. Fitzgerald *et al.*

An automated system of sample positioning and analysis was developed to be used with an Oceanography International Total Carbon System. This system analyses 25 samples/h, as compared to 10 samples/h using the manual method. The overall mean relative standard deviation (RSD) of the automated system was 3·7%. This is more precise than the 4·4% mean RSD of the manual method. The accuracy of the automated method, using reference samples supplied by the Florida Department of Environmental Regulation and chi-square analysis, was within 99·9% of the expected value. This compares favourably with previous reference samples analysed using the manual method. The basic operation of the automated system uses sequential logic, limit switches and relays to actuate air cylinders. The air cylinders position, seal and pierce the ampules containing the sample. Once pierced, the sample vial is purged of $CO_2$ produced during sample oxidation. The $CO_2$ is sent to an infra-red analyser where it is measured and a relative value recorded and stored in a data file.

### Un système automatique d'analyse d'échantillons pour un analyseur du carbone total

L. Fitzgerald *et al.*

Un système automatique pour le positionnement d'échantillons et leur analyse a été développé pour être utilisé avec un Système de Carbone Total d'Océanographie Internationale. Ce système analyse 25 échantillons par heure comparé à 10 échantillons par heure utilisant la méthode manuelle. L'écart type relatif (RSD) du système automatique était de 3,7%. Ceci est plus précis que l'écart type moyen de 4,4% de la méthode manuelle. L'exactitude de la méthode automatique utilisant des échantillons de références du Département du Florida de la Régulation environmental et de l'analyse Chi-Square était de 99,9% de la valeur prévue. Ceci se compare de façon favorable avec des échantillons de référence utilisés avec la méthode manuelle. Les opérations de base du système automatique utilisent une logique séquentielle des switches de limite et des relais qui activent des cylindres d'air. Les cylindres d'air positionnent, ferment et percent les ampoulles qui contiennent l'échantillon. Une fois percée, l'ampoulle contenant l'échantillon est purgée du $CO_2$ produit pendant l'oxidation de l'échantillon. Le $CO_2$ est envoyé à un analyseur infrarouge ou il est mesuré et une valeur relative est enregistrée et mise en mémoire dans un ordinateur.

### Ein automatisiertes System der Probenanalyse für einen Gesamtkohlenstoff-Analysator

L. Fitzgerald *et al.*

Für das Oceanography International Total Carbon System Gerät wurde ein automatisiertes System für die Probenpositionierung und Analyse entwickelt. Dieses System analysiert 25 Proben pro Stunde, verglichen mit 10 Proben pro Stunden bei manuellem Betrieb. Die alles umfassende mittlere relative Standardabweichung (RSA) des automatisierten Systems betrug 3,7%. Das ist genauer als die 4,4% mittlere RSA der manuellen Methode. Die Genauigkeit der automatisierten Methode bei Benützung von Referenzproben des Florida Department of Environmental Regulation und des Chi-Quadrat Tests war innerhalb von 99,9% des erwarteten Wertes. Das vergleicht sich gut mit früheren Referenzproben, die mit der manuellen Methode analysiert wurden. Die Grundoperationen des automatisierten Systems benützen sequentielle Logik, Grenzschalter und Relais zur Betätigung von Druckluftkolben. Die Druckluftkolben positionieren, dichten und durchstossen die Ampullen mit den Proben. Wenn geöffnet wird im Probengefäss das $CO_2$, das während der Oxydation entsteht, ausgespühlt. Das $CO_2$ wird zu einem Infrarotgerät geführt, wo es vermessen wird und der Relativwert notiert und in einer Datei gespeichert wird.

### Page 84

### Gamma photon attenuation measurement as a technique for monitoring liquid composition

S. Everett and D. J. Malcolme-Lawes

The attenuation of 60 keV gamma photons by liquid mixtures flowing through a 10 cm diameter pipe has been examined as a means of monitoring liquid compositions. It is observed that with a photon detection rate of about $10^4 s^{-1}$ a precision of about 4% for the percentage composition of a binary mixture may be obtained using measurement times of 10 s. The precision is expected to improve significantly through the use of a more intense gamma photon beam.

### La mesure de l'atténuation de photons gamma comme technique pour le contrôle de la composition de liquides

S. Everett and D. J. Malcolme-Lawes

L'atténuation de photons gamma de 60 keV par des mélanges liquides coulant à travers des tubes de 10 cm de diamètre a été examinée pour permettre le contrôle de composition de liquides. Il a été observé qu'avec une détection de 10 photons par seconde une précision d'environ 4% pour le pourcentage de la composition d'un mélange binair peut être obtenue utilisant un temps de mesure de 10 secondes. Il est prévu d'améliorer la précision de façon significative en utilisant un fésseau de photons gamma plus intense.

### Gammaphotonen-Attenuationsmessungen als Technik für die Überwachung der Zusammensetzung von Flüssigkeiten.

S. Everett and D. J. Malcolme-Lawes

Die Abschwächung von 60 keV Gammastrahlung durch flüssige Lösungen, die durch eine Röhre von 10 cm Durchmesser fliessen, wurde auf die Verwendung zur Überwachung flüssiger Mischungen untersucht. Es konnte beobachtet werden, dass mit einer Photonendetektionsgeschwindigkeit von $10^4 s^{-1}$ eine Präzision von etwa 4% für die prozentuale Zusammensetzung einer binären Mischung erhalten wird bei einer Messzeit von 10 s. Es wird erwartet, dass sich die Präzision bei Verwendung intensiverer Gammaphotonenstrahlen stark verbessert.

### Page 88

### Proposed international concept for instrument testing

R. Haeckel

Joint efforts of the IFCC's Expert Panel on Instrumentation and of the ECCL's *Ad Hoc* Committee on Instrument Testing have led to a three-stage concept for the evaluation of analytical instruments as, for example, analysers which are introduced into the market. These stages and the involvement of manufacturers and users are described.

### Suggestion pour un concept international pour tester des instruments

R. Haeckel

Des efforts conjoints des experts sur l'instrumentation de la IFCC et du Comité *Ad Hoc* de la ECCLS pour tester des instruments ont mené à un concept à trois paliers pour l'évaluation d'instruments analytiques tels que les analyseurs qui sont introduits sur le marché. Ces étappes et l'implication des producteurs et utilisateurs sont décrites.

### Vorschlag für ein internationales Konzept zur Geräteprüfung

R. Haeckel

Das Expert Panel on Instrumentation der IFCC und das *Ad Hoc* Committee on Instrument Testing des ECCLS haben gemeinsam ein Drei-Stadien Konzept zur Evaluation analytischer Geräte wie z. B. von Analysatoren, die in den Markt eingeführt werden sollen, erarbeitet. Die 3 Stadien sowie die Rolle von Hersteller und Anwender werden beschrieben.

### Page 91

### Decision theory approach to establishing an in-house histology laboratory in small hospitals

Ik-Whan Kwon *et al.*

Establishing a histology department in a small hospital causes a special problem due to the volume of specimens and cost of equipment. When such a decision is being made under the conditions of uncertainty with several choices, an integrated approach has to be employed which considers all options available to the decision-maker (laboratory director).

This study employed decision-theory analysis in reviewing and selecting an optimum decision among three mutually exclusive courses of action in a small hospital (95 beds) in the midwestern region of the USA; establishing an in-house histology department, using a reference laboratory, or using a partial reference laboratory. In integrating all monetary criteria into a single decision model, it was shown that the most important decision variable was the annual break-even volume of specimens.

Since the annual volume of specimens was lower than the required break-even volume, it was decided, and subsequently recommended to the Administration of the hospital, that an addition of histology department to the existing laboratory facilities was not justified. The final decision was made by the Administration based on the recommendation that the hospital should continuously use the reference laboratory system until the volume is sufficiently large to warrant adding an in-house histology department.

### Une approche par la théorie de décision pour établir un laboratoire d'histologie dans des petits hôpitaux

Ik-Whan Kwon *et al.*

Etablir un département d'histologie dans un petit hôpital cause des problèmes spéciaux dus au volume d'échantillons et dus au coût de l'équipement. Quand une telle décision doit être faite sous les conditions de l'incertitude de différents choix, un approche intégré doit être employé qui considère toutes les options accessibles à la personne qui prend la décision (directeur de laboratoire).

Cette étude a employé l'analyse de la théorie de décision pour étudier et choisir une décision optimale entre trois possibilités d'action mutuellement exclusives dans un petit hôpital (95 lits) dans une région de l'ouest des Etats-Unis; établir son propre département d'histologie ou utiliser un laboratoire de référence ou utiliser un laboratoire de référence partielle. En intégrant tous les critères financiers en un modèle de décision, il a pu être montré que la variable de décisions la plus importante était le nombre annuel d'échantillons.

Car le nombre annuel d'échantillons était inférieur au nombre requis pour la justification, il a été décidé et ensuite recommandé à l'administration de l'hôpital que d'ajouter un département d'histologie aux facilités de laboratoire existantes n'était pas justifié. La décision finale faite par l'administration s'est basée sur cette recommandation que l'hôpital va utiliser le système de laboratoire de référence tant que le nombre d'échantillons n'est pas suffisamment grand pour justifier son propre laboratoire d'histologie.

### Anwendung der Entscheidungstheorie auf die Einrichtung eines eigenen Histologie-Labors in kleinen Spitälern

Ik-Whan Kwon *et al.*

Die Einrichtung eines Histologie-Departements in einem Kleinspital verursacht spezielle Probleme wegen der Zahl der Proben und wegen der Kosten der Geräte. Wenn ein solcher Entscheid unter der Bedingung der Unsicherheit bezüglich verschiedener Möglichkeiten gefällt werden muss, so ist ein integraler Ansatz zu verwenden, der alle vorhandenen Optionen, die der Entscheidungsstelle (Laboratoriumsdirektor) offen stehen, berücksichtigt.

Diese Studie verwendet Entscheidungstheorie-Analyse bei der Begutachtung und Auswahl eines optimalen Entscheides zwischen drei sich gegenseitig ausschliessenden Aktionsplänen in einem Kleinspital (95 Betten) in der Region des mittleren Westens: Einrichtung eines eigenen Histologie-Departements, Benützung eines Referenz-Laboratoriums, oder teilweise Benützung eines Referenz-Laboratoriums. Unter Einbezug aller finanziellen Kriterien in ein einziges Entscheidungsmodell wird gezeigt, dass die wichtigste Entscheidungsvariable die jährliche Break-even Zahl der Proben ist.

Weil die jährliche Probenzahl unter dem Break-even Punkt lag, wurde entschieden und entsprechend der Spitaladministration empfohlen, dass sich die Erweiterung der bestehenden Laboratoriumseinrichtungen um ein Histologie-Departement nicht rechtfertigen lässt. Der endgültige Entscheid der Administration basierte auf dieser Empfehlung, dass das Spital weiterhin ein Referenz-Laboratoriumssystem benützen werde bis die Probenzahl genügend gross ist, um die Anfügung eines eigenen Histologie-Departement zu rechtfertigen.

### Page 94

### Carry-over in automatic analysers
P. M. G. Broughton

A method is described for the measurement of reagent carry-over, which can arise in some types of selective analyser where different tests are performed in sequence on the same specimen. The effect of both sample and reagent carry-over on the design of experiments for the measurement of imprecision is considered. It is suggested that within-batch imprecision should be measured with a series of replicate samples for the same test. This is the optimal arrangement, where there are no effects from sample or reagent carry-over. The design used to measure between-day imprecision should include all random errors, due to both types of carry-over, which could affect the analytical results of patients' specimens.

### La contamination dans les analyseurs automatiques
P. M. G. Broughton

Une méthode est décrite pour la mesure de la contamination des réactifs qui peut se produire dans certains types d'analyseurs sélectifs qui permettent de faire plusieurs tests en séquence sur le même échantillon. L'effet de la contamination par l'échantillon et le réactif sur l'expérience pour la mesure de l'imprécision est pris en considération. Il est suggéré que l'imprécision à l'intérieur d'une série doit être mesurée avec une série d'échantillons identiques pour le même test. Ceci est l'arrangement optimal qui ne produit aucun effet par la contamination de l'échantillon ou du réactif. L'expérience utilisée pour mesurer l'imprécision entre les jours doit inclure toutes les erreurs dues au hasard et dues aux deux types de contamination qui peuvent affecter le résultat analytique des échantillons des malades.

### Verschleppungseffekte in Analysenautomaten
P. M. G. Broughton

Es wird eine Methode beschrieben für die Bestimmung der Reagensverschleppung, welche in gewissen Typen von selektiven Analysenautomaten auftreten kann, bei denen hintereinander verschiedene Analysen an der gleichen Probe durchgeführt werden. Die Wirkung von Proben- und Reagensverschleppung auf die Planung von Experimenten zur Bestimmung der Ungenauigkeit wird diskutiert. Es wird vorgeschlagen, dass die Präzision innerhalb einer Serie mit einer Reihe gleicher Proben für den gleichen Test gemessen werden sollte. Dies ist die optimale Anordnung, wo keine Effekte der Verschleppung von Probe oder Reagens auftreten können. Die Planung für die Bestimmung der Präzision von Tag zu Tag sollte alle zufälligen Fehler einschliessen, die auf beide Typen der Verschleppung, welche das Analysenresultat einer Patientenprobe beeinflussen könnten, zurückzuführen sind.

---

The following should have been published in *JAC*, Volume 6, Number 1 (January–March 1984), we apologize for the delay:

### Microprocessors in pathology
F. Murphy

This is one of the papers read at the meeting on the Application of Microprocessors in Hospital Practice held on 25 May 1983 by the UK Liaison Committee for Sciences Allied to Medicine & Biology, London.

The author presents the role of the microprocessor, in distinction from the mini or mainframe computer, in pathology. The main areas of application are described and personal experiences of microprocessor implementation in the author's laboratory. These include the BBC microcomputer interfaced to the Gilford 203 System Clinical Analyser and to the Technicon SMA II.

This paper was first published in *Journal of Medical Engineering & Technology* (Taylor & Francis Ltd, London).

### Utilisation de microprocesseurs en pathologie
F. Murphy

Ceci est une des présentations faite lors de la rencontre sur l'application de microprocesseurs dans les hôpitaux tenue le 25 mai 1983 par l'Association pour les sciences appliquées à la médecine et biologie à Londres. L'auteur présente le rôle du microprocesseur, en distinction du mini ou grand ordinateur, en pathologie. Les domaines principaux d'application sont décrits et les expériences personnelles avec l'implémentation de microprocesseurs dans le laboratoire de l'auteur. Ces expériences incluent le microordinateur BBC interfacé à l'analyseur clinique Gilford 203 et au Technicon SMA II.

### Mikroprozessoren in der Pathologie
F. Murphy

Dies ist eine Arbeit, die am 25. Mai 1983 an der Konferenz 'Application of Mikroprocessors in Hospital Practice' der Association for Sciences Allied to Medicine & Biology in London vorgetragen wurde.

Die Autoren stellen die Rolle des Mikroprozessors in der Pathologie im Gegensatz zu der des Mini- oder Grossrechners dar. Die Hauptanwendungsgebiete und die persönlichen Erfahrungen des Mikroprozessor-Einsatzes im Laboratorium des Autors werden beschrieben. Diese umfassen unter anderem den Anschluss eines BBC Mikrorechners an den Gilford 203 System Clinical Analyser und den Technicon SMA II.

### FORTHCOMING PAPERS

*The following will be included in later 1984 issues.*

A microprocessor data-capture and database package for the IL 508 analyser
*B. Clark et al.*

Experiences with a system for signal- and data-processing, together with on-line variance reduction, in continous-flow analysers
*R. Maasen and H. Baadenhuijsen*

A flexible computer-computer protocol for DISNET
*P. J. Gemperline and R. Megargle*

Erythrocyte transketolase activity measured on the Gemstar discrete analyser
*J. E. Buttery et al.*

A computer program for intra-laboratory quality control
*C. Zuppi et al.*

Automated enzymatic determination of plasma-free fatty acids by centrifugal analysis
*D. P. Knox and D. G. Jones*

A microcomputer-based automated curve tracer for accurate digitization of paper-recorded spectra
*J. C. W. G. Bink and H. A. Van't Klooster*

The evaluation kit for clinical chemistry: a practical guide for the evaluation of methods, instruments and reagent kits
*G. H. White and C. G. Fraser*

A microcomputer system for use with an EPR spectrometer
*M. J. Adams and G. J. Ewen*

Turbidimetric analysis on the Hitachi 705 using orosmucoid as a model
*E. F. Legg and M. Sullivan*

The Abbott TDX evaluated for T-uptake
*G. A. Harff*

